# The Epidemiology of Murder-Suicide in the US, 2016-2022

**DOI:** 10.1001/jamanetworkopen.2025.23698

**Published:** 2025-07-29

**Authors:** Katherine M. Keyes, Victoria A. Joseph, Caroline Rutherford

**Affiliations:** 1 Department of Epidemiology, Columbia University, New York, New York

## Abstract

This cross-sectional study examines rates and characteristics of murder-suicides in the US between 2016 and 2022.

## Introduction

Murder-suicide events occur when an individual commits 1 or more acts of homicide shortly before taking their own life.^1,2^ Incidence is low in the US population, with prior research indicating stable population rates of approximately 0.2 to 0.3 per 100 000,^3^ although incidence data were not included after 2004. Given dynamic trends in suicide and homicide, a more recent assessment of incidence, characteristics, and trends is important for informing areas for potential prevention and intervention for those at risk. The present study estimated murder-suicide rates and characteristics in 30 US states from 2016 through 2022.

## Methods

Data for this repeated cross-sectional time-series (2016-2022) were drawn from the National Violent Death Reporting System Restricted Access Database,^4^ including 30 states that contributed data consistently (eMethods in Supplement 1). Briefly, events were based on police reports and other information regarding whether 1 or more homicides were followed by suspect suicide within a 24-hour time period. Data on each incident, including the suicide decedent and all linked homicides linked, were extracted for analysis. Rates were estimated with US Census Bureau total state population denominators. Piecewise linear regression was used to statistically evaluate time trends in murder-suicide rates by quarter and year.

Data were accessed through an approved data use agreement; analyses were approved by the Columbia University institutional review board. This study followed the Strengthening the Reporting of Observational Studies in Epidemiology (STROBE) reporting guideline for cross-sectional studies. Data were analyzed with SAS version 9.4 (SAS Institute Inc) and R version 4.2.1 (R Project for Statistical Computing).

## Results

From 2016 through 2022 in the 30 states with complete reporting, 5743 deaths were involved in murder-suicide events (an average of 820 deaths per year), including 3125 homicide and 2618 suicide decedents (2398 male [91.6%]; 540 Black [20.6%], 1851 White [70.7%]) (Table).

**Table.** Characteristics of Murder-Suicides in the US, 2016-2022

Characteristic	Decedents, No. (%)	
	Homicide (n = 3125)	Suicide (n = 2618)
Sex		
Male	2289 (73.3)	2398 (91.6)
Female	836 (26.8)	220 (8.4)
Age, y		
<18	433 (13.9)	23 (0.9)
18-34	794 (25.4)	679 (25.9)
35-64	1356 (43.4)	1455 (55.6)
≥65	542 (17.34)	461 (17.6)
Race		
Black or African American	535 (17.1)	540 (20.6)
White	2279 (72.9)	1851 (70.7)
Other or unknown^a^	311 (10.0)	227 (8.7)
Marital status		
Married, civil union, domestic partnership, or separated	1295 (41.4)	1036 (39.6)
Never married	1183 (37.9)	786 (30.0)
Divorced	433 (13.9)	413 (15.8)
Widowed	167 (5.3)	313 (12.0)
Single or unknown	47 (1.5)	70 (2.7)
Primary weapon in the homicide		
Firearm	2775 (88.9)	2373 (90.7)
Sharp instrument	152 (4.9)	67 (2.6)
Hanging, strangulation, suffocation	66 (2.1)	67 (2.6)
Other	132 (4.1)	110 (4.2)
Homicide decedents’ relationships to suspect		
Child	374 (12.5)	NA
Ex-girlfriend or ex-boyfriend	167 (5.6)	
Girlfriend or boyfriend	481(16.1)	
Parent	181 (6.1)	
Spouse	982 (32.9)	
Ex-spouse	77 (2.6)	
Friend	47 (1.6)	
In-law	35 (1.2)	
Other family member	47 (1.6)	
Other person, known to person murdered	143 (4.8)	
Relationship unknown	104 (3.5)	
Sibling	57 (1.9)	
Stranger	44 (1.5)	
Other	244 (8.2)	
No. of homicide decedents per murder-suicide incident		
1	2240 (85.8)	NA
2	274 (10.5)	
3	69 (2.6)	
≥4	27 (1.0)	

Abbreviation: NA, not applicable.

^a^ American Indian or Alaska Native, Asian, Native Hawaiian or Other Pacific Islander, 2 or more races, and unknown.

The overall murder-suicide rate from 2016 to 2022 for the 30 states included in this analysis was 0.45 per 100 000 persons, with annual rates from 0.44 per 100 000 persons in 2016 to 0.50 per 100 000 persons in 2022. By quarter, rates ranged from 0.09 per 100 000 persons (July-September 2021) to 0.13 per 100 000 persons (July-September 2022). The Figure shows the trend over time by quarter; the best fitting model was a single slope (β = 0.0004 [SE, 0.0003]; *P* = .15), indicating no significant changes. The rate of murder-suicide varied more than 5-fold between states; rates were highest in Alaska (0.87 per 100 000 persons) and Arizona (0.70 per 100 000 persons), and lowest in Massachusetts (0.20 per 100 000 persons) and New Hampshire (0.16 per 100 000 persons).

**Figure.**
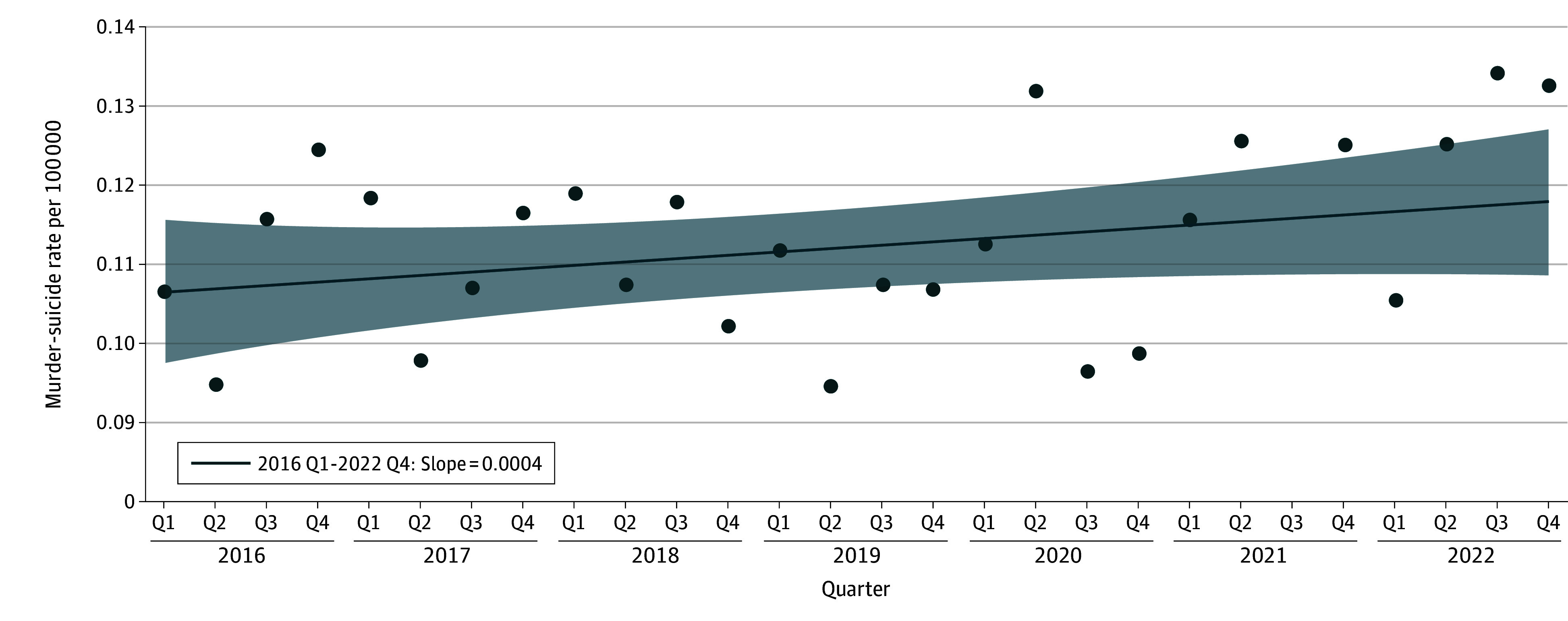
Murder Suicide Rate in the US Per 100 000 Persons by Quarter (Q), 2016-2022 Piecewise regression analysis indicated that the best fitting model included 1 linear slope (0 permutations); the magnitude of the slope was β = 0.0004 (SE, 0.0003) (*P* = .15). Furthermore, this figure shows the trend over time in the murder-suicide rate in the US, with a fitted regression line along with 95% CIs (shaded area). Dots represent observed data points for each month.

The number of homicide decedents per incident ranged from 1 to 7, with 2240 events (85.8%) involving 1 homicide decedent, 274 (10.5%) involving 2, and 96 (3.7%) involving 3 or more (Table). Male individuals represented 2398 of 2618 suicide decedents (91.6%) and 2289 of 3125 homicide decedents (73.2%). Among homicide decedents, 433 (13.9%) were children under 18 years. A total of 2775 homicides (88.9%) and 2373 suicides (90.7%) involved a firearm as the primary weapon. Deaths between romantic partners were most frequent; 1707 homicide decedents (57.2%) were the former or current romantic partner of the perpetrator.

## Discussion

In this repeated cross-sectional study of murder-suicide rates in 30 US states, the annual incidence of murder-suicide was higher than previously reported estimates,^3^ suggesting that while rare, murder-suicide events are occurring more frequently than earlier research indicated. From 2016 to 2022, murder-suicide rates remained relatively stable. Given that over half of homicide decedents in murder-suicides were current or former intimate partners of the perpetrator, strengthening domestic violence screening and intervention programs^5^ should be prioritized. The nearly 90% involvement of firearms suggests that policies and interventions targeting firearm access, including through extreme risk protection orders,^6^ could be particularly effective in preventing these events. The substantial geographic variation in murder-suicide rates indicates a need for state-specific prevention approaches that account for local gun policies, mental health resources, and domestic violence response systems. Limitations of the study include that not all states in the US contributed data consistently through the study period, thus results are not nationally representative, and that circumstance information is not available for all cases.

## Notes

Supplement 1.
eMethods.

Supplement 2.
Data Sharing Statement
