# Supplementary material for: The Epidemiology of Murder-Suicide in the US, 2016-2022
Source: JAMA Netw Open. 2025 Jul 29;8(7):e2523698. doi: 10.1001/jamanetworkopen.2025.23698 (PMC12308426; doi:10.1001/jamanetworkopen.2025.23698)
Supplement: Supplement 1. — eMethods. [file jamanetwopen-e2523698-s001.pdf]

Supplemental Online Content

Keyes KM, Joseph VA, Rutherford C. The epidemiology of murder-suicide in the United States, 2016-2022. *JAMA Netw Open.* 2025;8(7):e2523698. doi:10.1001/jamanetworkopen.2025.23698

eMethods.

This supplemental material has been provided by the authors to give readers additional information about their work.

© 2025 Keyes KM et al. *JAMA Network Open*.

Supplemental methods section

Data for this repeated cross-sectional time-series were drawn from annual data from the CDC's National Violent Death Reporting System Restricted Access Database (NVDRS RAD).[1] The NVDRS RAD is a comprehensive, multi-state surveillance dataset that provides detailed individual-level information on violent deaths, combining data from death certificates, coroner and medical examiner reports, toxicology tests, and law enforcement reports. The NVDRS RAD is a comprehensive, multi-state surveillance dataset that provides detailed individual-level information on violent deaths, combining data from death certificates, coroner and medical examiner reports, toxicology tests, and law enforcement reports. NVDRS-RAD data are abstracted from source documents following standardized coding developed by the CDC, and validated through the NVDRS software and manual reabstraction. Annual quality control checks are conducted by CDC to ensure high reliability of the data.[2]

This analysis used data from 2016 to 2022, and included the 30 US states that contributed data consistently over the study period. The states included in the analysis are drawn from all four census regions (Midwest, Northeast, South, and West), and at least one state from all 9 census divisions. From the Midwest, included states were: Illinois, Indiana, Michigan, Ohio, Wisconsin, Iowa, Kansas, Minnesota. From the Northeast, states were New Jersey, Pennsylvania, Connecticut, Maine, Massachusetts, New Hampshire, Rhode Island, and Vermont. From the South, states were Kentucky, Georgia, Maryland, North Carolina, South Carolina, Virginia, and Oklahoma. From the West, states were Arizona, Colorado, New Mexico, Utah, Alaksa, Oregon, and Washington.

Data were accessed through an approved data use agreement; analysis plans were approved by the Columbia University Institutional Review Board. Strengthening the Reporting of Observational Studies in Epidemiology (STROBE) guidelines were followed.

Murder-suicide. Murder-suicide events were coded in NDVRS RAD based on police reports and other information such as death certificates, coroner and medical examiner records regarding whether one or more homicides were followed by suspect suicide within a 24-hour time period. Data on each death associated with the incident, including the suicide decedent and all homicides linked to them, were extracted for analysis. Primary weapon in each victim's death, homicide decedents' relationship to suspect, and the number of homicide decedents per murder-suicide incident were included in each study record base on available records.

By quarter-year, the count of murder-suicide events ranged from 164 in April-June 2016 to 239 in July-September 2022.

Additional variables.
Race category. Victim and suspect race were included in the study record. Race classifications were abstracted by trained data abstractors from primary source documents, including death certificates, coroner/medical examiner reports, and law enforcement reports, according to standards set by the U.S. Department of Health and Human Services (HHS) and the Office of Management and Budget (OMB). Originally, race was categorized as American Indian or Alaska Native, Asian, Black or African American, Native Hawaiian or Other Pacific Islander, Two or More Races, White, and Unknown. Due to data sparsity, these categories were recoded for this analysis into Black or African American, White, and Other/Unknown, where 'Other/Unknown' includes American Indian or Alaska Native, Asian, Native Hawaiian or Other Pacific Islander, Two or More Races, and Unknown.

Additional demographics. Each homicide and suicide decedent's sex, age, and marital status were extracted from the NVDRS record. Sex was categorized as male or female; age categorized as <18, 18-

© 2025 Keyes KM et al. *JAMA Network Open*.

34, 35-64, and 65+, and marital status categorized as Married/Civil Union/Domestic Partnership/Separated, never married, divorced, widowed, and single/unknown.

Statistical analysis.

We first examined the distributions of demographics and characteristics of murder-suicide events. Then, rates of murder-suicide were estimated with US Census Bureau total state population denominators. Piecewise linear regression[3] was used to statistically evaluate time trends in murder-suicide rates, estimating whether there are significant changes in trends by fitting a series of connected linear segments. We used quarter-years as time points, dividing each year into four quarters (January-March, April-June, July-September, October-December). Piecewise linear regression approach uses permutation tests to determine the optimal number and placement of breaks in the linear time series—points in time where significant changes in slope occur—thus providing annual percent changes (APCs) and average annual percent changes (AAPCs) to quantify trends over the study period.

© 2025 Keyes KM et al. *JAMA Network Open*.

1. Centers for Disease Control and Prevention. National Violent Death Reporting System (NVDRS). Published 2025. Accessed September 6, 2024. https://www.cdc.gov/nvdrs/about/index.html
2. CDC. National Violent Death Reporting System Web Coding Manual Version 6.1. Accessed May 23, 2025. https://www.cdc.gov/violenceprevention/datasources/nvdrs/resources.html
3. Joinpoint Regression Program. Statistical Methodology and Applications Branch, Surveillance Research Program, National Cancer Institute. Published 2025. Accessed May 23, 2025. https://surveillance.cancer.gov/joinpoint/

© 2025 Keyes KM et al. *JAMA Network Open*.
