# Supplementary material for: The Epidemiology of Murder-Suicide in the US, 2016-2022
Source: JAMA Netw Open. 2025 Jul 29;8(7):e2523698. doi: 10.1001/jamanetworkopen.2025.23698 (PMC12308426; doi:10.1001/jamanetworkopen.2025.23698)
Supplement: Supplement 2. — Data Sharing Statement [file jamanetwopen-e2523698-s002.pdf]

Data Sharing Statement

Keyes. The Epidemiology of Murder-Suicide in the US, 2016-2022. *JAMA Netw Open*. Published July 29, 2025. doi:10.1001/jamanetworkopen.2025.23698

Data
Data available: No

Additional Information
Explanation for why data not available: Data are available from NCHS through data use agreements
